# Gene-expression analysis of a colorectal cancer-specific discriminatory transcript set on formalin-fixed, paraffin-embedded (FFPE) tissue samples

**DOI:** 10.1186/s13000-015-0363-4

**Published:** 2015-07-25

**Authors:** Alexandra Kalmár, Barnabás Wichmann, Orsolya Galamb, Sándor Spisák, Kinga Tóth, Katalin Leiszter, Boye Schnack Nielsen, Barbara Kinga Barták, Zsolt Tulassay, Béla Molnár

**Affiliations:** 2nd Department of Internal Medicine, Semmelweis University, Budapest, Hungary; Molecular Medicine Research Unit, Hungarian Academy of Sciences, Budapest, Hungary; Bioneer A/S, Hørsholm, Denmark; 2nd Department of Medicine Semmelweis University, Szentkirályi str. 46., 1088 Budapest, Hungary

**Keywords:** Colorectal cancer, Gene expression, qRT-PCR, In situ hybridization, Fresh frozen, Formalin-fixed, Paraffin-embedded

## Abstract

**Background:**

A recently published transcript set is suitable for gene expression-based discrimination of normal colonic and colorectal cancer (CRC) biopsy samples. Our aim was to test the discriminatory power of the CRC-specific transcript set on independent biopsies and on formalin-fixed, paraffin-embedded (FFPE) tissue samples.

**Methods:**

Total RNA isolations were performed with the automated MagNA Pure 96 Cellular RNA Large Volume Kit (Roche) from fresh frozen biopsies stored in RNALater (CRC (n = 15) and healthy colonic (n = 15)), furthermore from FFPE specimens including CRC (n = 15) and normal adjacent tissue (NAT) (n = 15) specimens next to the tumor. After quality and quantity measurements, gene expression analysis of a colorectal cancer-specific marker set with 11 genes (*CA7, COL12A1, CXCL1, CXCL2, CHI3L1, GREM1, IL1B, IL1RN, IL8, MMP3, SLC5A7*) was performed with array real-time PCR using Transcriptor First Strand cDNA Synthesis Kit (Roche) and RealTime ready assays on LightCycler®480 System (Roche). In situ hybridization for two selected transcripts (*CA7, CXCL1*) was performed on NAT (n = 3), adenoma (n = 3) and CRC (n = 3) FFPE samples.

**Results:**

Although analytical parameters of automatically isolated RNA samples showed differences between fresh frozen biopsy and FFPE samples, both quantity and the quality enabled their application in gene expression analyses. CRC and normal fresh frozen biopsy samples could be distinguished with 93.3 % sensitivity and 86.7 % specificity and FFPE samples with 96.7 and 70.0 %, respectively. In situ hybridization could confirm the upregulation of *CXCL1* and downregulation of *CA7* in colorectal adenomas and tumors compared to healthy controls.

**Conclusion:**

According to our results, gene expression analysis of the analyzed colorectal cancer-specific marker set can also be performed from FFPE tissue material. With the addition of an automated workflow, this marker set may enhance the objective classification of colorectal neoplasias in the routine procedure in the future.

**Electronic supplementary material:**

The online version of this article (doi:10.1186/s13000-015-0363-4) contains supplementary material, which is available to authorized users.

## Background

Colorectal cancer (CRC) is one of the leading malignant neoplasms worldwide, and early diagnosis and molecular characterization is considered essential to decrease CRC-related deaths [1]. A discriminatory set of transcripts was recently identified that proved to be suitable for distinguishing CRC from normal colon with high sensitivity and specificity [2]. The study used 53 biopsies fresh frozen colon samples along the dysplasia-carcinoma transition containing CRC (n = 22), adenoma (n = 20) and 11 healthy colon (n = 11) specimens, and a qPCR-based expression profile (transcript set) was identified that discriminated the malignant from benign colon.

Although the best specimens for gene expression analysis are fresh frozen tissue samples stored with or without stabilization reagents (e.g. RNA Stabilization Reagent) with highly intact RNA content, difficulties and disadvantages of fresh frozen specimen collection (as requiring special logistic issues, equipment and hands-on time) can limit the number of fresh frozen samples in certain hospitals. In parallel, formalin-fixed, paraffin-embedded (FFPE) tissue samples are routinely collected for diagnostic purpose and are stored at room temperature in archives for several years. Diagnosis of colorectal diseases are commonly made on the basis of histology of stained slides from FFPE biopsies or bigger removed tissue samples taken during routine endoscopy examination or surgery. The fixation in formalin conserves the tissue structure, but holds technical disadvantages in terms of molecular sample quality, such as nucleic acid degradation and crosslink formation; this renders it a sub-optimal sample source [3, 4]. In order to optimize sample quality from FFPE tissue samples, manual and automated nucleic acid isolation kits and FFPE-optimized protocols are continuously being developed, that can also enhance the use of FFPE samples in gene expression studies, as well [5–7]. Automated nucleic acid isolation systems hold the possibility to remarkably increase the throughput of diagnostic laboratories. Furthermore, automated protocols have the advantage of standardization, minimal hands-on time that is crucial in studies with high sample numbers [8].

Although molecular characterization of homogenized tissue samples serves information about the whole tumor mass, up- or downregulation of genes can be characteristic for some certain tumor areas or cell types [9]. In situ analysis can broaden our knowledge by providing information about tumor heterogeneity, as well as the localization of the altered mRNA expression.

As a validation study, we aimed to extend the analysis of the previously published CRC-specific marker set on FFPE samples. The applicability of this marker set was tested on 30 independent fresh frozen biopsy samples (CRC: n = 15; healthy colon: n = 15) and also on 30 formalin-fixed, paraffin-embedded (FFPE) tissue samples (CRC: n = 15; healthy colon: n = 15) after RNA isolation with an automated method. Furthermore, in situ hybridization was performed on 9 FFPE samples (CRC: n = 3; adenoma: n = 3; healthy colon: n = 3).

## Methods

### Patients and sample collection

Biopsy samples (approx. 5 mg tissue) were collected during routine endoscopy from patients scheduled for screening colonoscopy including untreated colorectal cancer (CRC) cases and healthy donors (n = 30; 15 CRC, 15 N). Biopsies were snap frozen and stored in RNALater at -80 °C. Independent formalin-fixed, paraffin-embedded (FFPE) tissue blocks of surgically removed CRC (UICC stage II-III) and corresponding normal adjacent (NAT) tissue specimens (n = 30; 15 CRC, 15 NAT) from the same patients were collected from a regional pathology archive. For in situ hybridization analysis NAT (n = 3), adenoma (n = 3) and CRC (n = 3) FFPE samples were used. No more than 6 months old FFPE blocks were used for RNA isolation. Written informed consent was obtained from all patients and donors. The study was approved by the local ethics committee (Semmelweis University Regional and Institutional Committee of Science and Research Ethics; Nr.: ETT TUKEB 23970/2011).

### RNA isolation

Fresh frozen biopsy samples in RNALater were thawed on ice and were transferred into MagNALyser Green Bead tubes containing ceramic beads and 800 μl MagNA Pure LC RNA Isolation Tissue Lysis Buffer (Roche Applied Science, Penzberg, Germany). All of the colorectal cancer samples contained tumor cell percentage higher than 80 %. No macrodissection was made prior to RNA isolations. Tissue samples were homogenized using MagNALyser instrument with 6500 rpm for 50 s twice. Ten micrometer thick sections were cut from the FFPE blocks; and each section was transferred into a microcentrifuge tube. Deparaffinization was performed with 1 ml xylene, incubating twice for 10 min, and 1 ml absolute ethanol also incubating twice for 10 min. Total RNA isolation was performed from 350 μl biopsy lysates and from each air dried deparaffinized section with the automated MagNA Pure 96 Cellular Large Volume Kit (Roche) on the MagNA Pure 96 nucleic acid isolation system according to the manufacturer’s instructions.

### Quantitative and qualitative analysis of the isolated RNA samples

RNA concentration and purity ratios (OD260/280, OD260/230) were measured with NanoDrop 1000 spectrophotometer (Thermo Fisher Scientific Inc., Waltham, USA). RNA quality was assessed using the RNA Integrity Number (RIN) measured by RNA 6000 Pico LabChip kit on a microcapillary electrophoresis system (Agilent BioAnalyzer 2100).

### Real-time PCR analysis

Using the Transcriptor First Strand cDNA Synthesis Kit (Roche Diagnostics), 150 ng of total RNA was reverse transcribed with the combination of anchored-oligo(dT) and random hexamer primers included in the kit (Cat no: 04897030001). Gene expression analysis was performed for eleven colorectal cancer specific markers [2] (CA7, COL12A1, CXCL1, CXCL2, CHI3L1, GREM1, IL1B, IL1RN, IL8, MMP3, SLC5A7) and 18S ribosomal RNA endogenous control (Table 1). RealTime ready assays from Universal Probe Library (Roche Applied Science) were used with forward and reverse primers (400 nM) and fluorescently labeled hydrolysis probes (200 nM) lyophilized into 384 well plates (Table 1). Real-time polymerase chain reaction analysis were performed in a final volume of 10 μl using 5 ng cDNA/well and 5 μl LightCycler® 480 Probes Master. The reagents and the samples were pipetted by epMotion 5070 liquid handling robot (Eppendorf). Thermal cycling conditions on the LightCycler® 480 System were the following: enzyme activation: 95 °C for 10 min, 45 cycles of amplification: 95 °C for 10 s, 60 °C for 30 s and signal detection at 72 °C for 1 s and cooling at 40 °C for 30 s. For data normalization 18S endogenous control was used.

**Table 1:** CRC-specific transcript set [2]

Gene symbol	Gene name	Amplicon length	RealTime ready assay ID (Roche)
CA7	carbonic anhydrase VII	77	103015
CHI3L1	chitinase 3-like 1	76	103035
COL12A1	collagen, type XII, alpha 1	66	103045
CXCL1	chemokine (C-C-C motif) ligand 1	105	105522
CXCL2	chemokine (C-C-C motif) ligand 2	95	103070
GREM1	gremlin 1	111	103109
IL1B	interleukin 1, beta	87	100950
IL1RN	interleukin 1 receptor antagonist	76	103133
IL8	interleukin 8	92	103136
MMP3	matrix metallopeptidase 3	110	103167
SLC7A5	solute carrier family 7, member 5	72	103210
RN18S1	RNA, 18S ribosomal 1, 18S ribosomal RNA	73	104092

For receiver operating characteristic (ROC) curve analysis, MedCalc13.3 software was applied to evaluate the discriminatory power of the examined genes. Interactive dot diagrams represent differences on a scale and indicate specificity and sensitivity values of the analyzed markers. For discriminant analysis SPSS 22.0 software was applied.

### In situ RNA hybridization

In situ analysis was performed on two of the eleven markers. CA7 was selected as it was the only downregulated transcript along CRC formation, while among the upregulated transcripts CXCL1 was included in the experiment, as it showed remarkable protein expression level changes between normal and CRC samples [10]. For both human CA7 mRNA (NM_005182.2) and CXCL1 mRNA (NM_001511.2), two non-overlapping oligonucleotide sequences were identified: for CA7 mRNA: 5’-TGGCATTCCAGTGAACCAGAT-3’(nucleotides 643-623) and 5’-AGCGCATCTGTCAGACGATTCAT-3’ (nucleotides 758-736), and for CXCL1 mRNA: 5’-ATGCAGGATTGAGGCAAGCTTT-3’ (nucleotides 347-326) and 5’-TTGGATTTGTCACTGTTCAGCAT-3’ (nucleotides 399-377). Locked nucleic acid (LNATM) oligonucleotides were designed resulting in RNA Tm’s of 87 °C, 84 °C, 85 °C, 86 °C, respectively. In addition, we included a negative control probe: Scramble 22’mer LNA oligo (RNA Tm 87 °C), and a positive control LNA probe against miR-126 (21’mer, RNA Tm 84 °C), see Jørgensen and collaborators, 2011 [11]. All LNA oligonucleotides were 6-carboxyfluorescein (FAM)-labeled at the 5’- and 3’-ends (double-FAM labeled probes) and obtained from Exiqon, Vedbæk, Denmark. In situ hybridization was performed using a TecanGenepaint in situ hybridization instrument (Tecan, Männedorf, Switzerland) essentially as described elsewhere [12]. In brief, 6 μm thick tissue sections from normal colon, colon adenomas and cancers, were deparaffinized and pretreated with proteinase-K (25 μg/ml for 8 min at 37 °C). In situ hybridization was performed by incubating the two double-FAM labeled CA7 and CXCL1 LNA probes mixed at 60 nM diluted in Exiqon hybridization buffer (Exiqon, Vedbæk, Denmark) at 57 °C for 1 h. Double-FAM labeled scramble (60 nM) and miR-126 (at 30 nM) probes were used as negative and positive controls, respectively. After stringent washes in SSC buffers, the sections were incubated with alkaline phosphatase–conjugated anti-FAM (1:800, Roche, Mannheim, Germany). Slides were developed in 4-nitro-blue tetrazolium (NBT) and 5-brom-4-chloro-3’-Indolyl-phosphate (BCIP) substrate (Roche) for 90 min resulting in a dark-blue precipitate. Slides were counter stained with Nuclear Fast Red (Vector Laboratories, Burlingname, CA). The slides were examined from digital whole slides obtained with 3DHISTECH scanner using a 20x objective.

## Results

### Quantity and quality of the isolated RNA samples

**Fig. 1:**
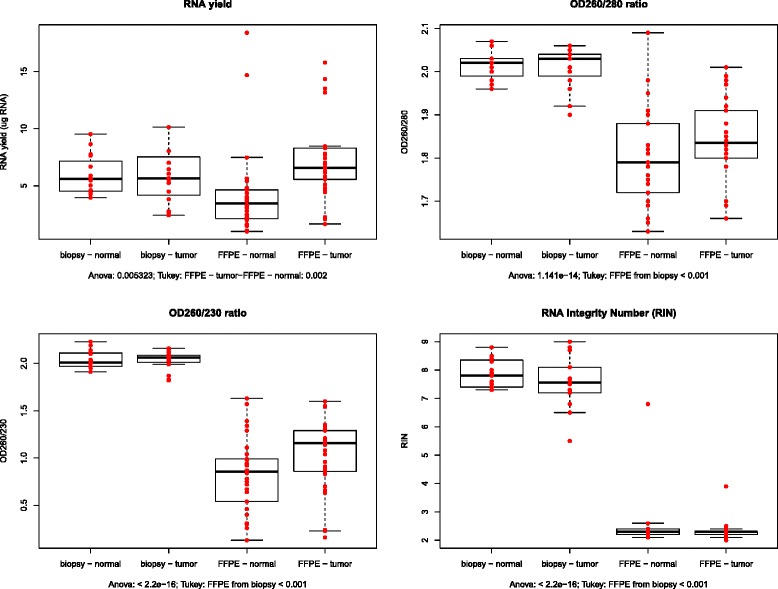
Analytical parameters of the automatically isolated RNA samples. **a** RNA yield (μg RNA); **b**) OD260/280; **c**) OD260/230 and **d**) RNA integrity number (RIN) of normal and tumor fresh frozen biopsy and FFPE samples. Individual quality or quantity measurements are represented by dots on the boxplots, while boxes indicate median and standard deviation of the data

We first isolated total RNA from 30 fresh frozen colonic endoscopy samples and from 30 FFPE resection samples with MagNA Pure 96 automated protocol and the yields, purity and integrity were compared. Yields of RNA isolated from fresh frozen samples and FFPE sections were found to be similar (Mean ± SD; fresh frozen: normal = 5.98 ± 1.72 μg, tumor = 5.77 ± 2.27 μg FFPE: normal = 4.20 ± 3.70 μg, tumor = 7.10 ± 3.30 μg) (Fig. 1a). Both OD260/280 and OD260/230 ratios were significantly higher (p < 0.001) in fresh frozen tissue samples compared to FFPE samples (Fig. 1b, 1c). Thus, as expected, the RNA purity ratios were found to be lower in RNA eluates isolated from FFPE samples than from frozen samples. RNA Integrity Numbers (RIN) were automatically generated by the software algorithm on the basis of the sample’s electropherogram and indicates the integrity of the total RNA on a scale from 1 to 10. The RINs were found to be significantly higher in the fresh frozen biopsy samples (normal = 7.87 ± 0.5; tumor = 7.55 ± 0.94) compared to FFPE samples (normal fresh frozen biopsy = 2.60 ± 1.20; tumor fresh frozen biopsy = 2.40 ± 0.50), (p < 0.001) (Fig. 1d).

### Gene expression analysis

**Fig. 2:**
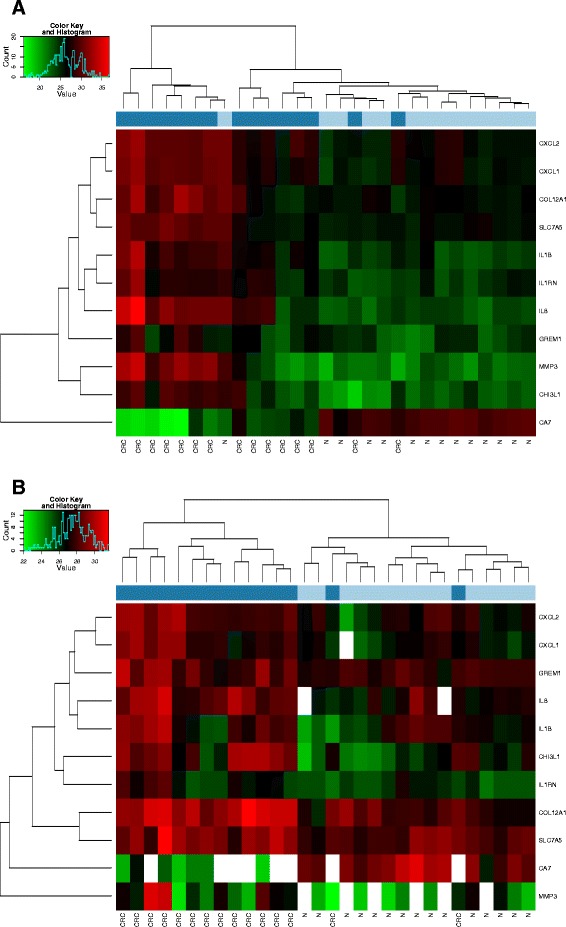
Heat maps of real-time PCR data representing gene expression alteration of the 11 analyzed transcripts in (**a**) fresh frozen biopsyand (**b**) FFPE samples. Color scale encodes relative overexpression (red) to underexpression (green)

Gene expression analysis of eleven CRC-specific markers was performed in order to test the performance of the isolated samples in real-time PCR reactions. Unsupervised hierarchical clustering revealed two major clusters in fresh frozen biopsy samples, one cluster with fresh frozen CRC cases with one misclassified normal case and another major cluster containing normal (14/15) and misclassified CRC cases (8/15) (Fig. 2a). In contrast, an almost clear separation of formalin-fixed, paraffin embedded colorectal cancer and normal colonic FFPE sample clusters could be found, only two CRCs were misclassified in the normal cluster (Fig. 2b).

### Discriminatory power of the marker set on independent fresh frozen biopsy and FFPE samples

**Fig. 3:**
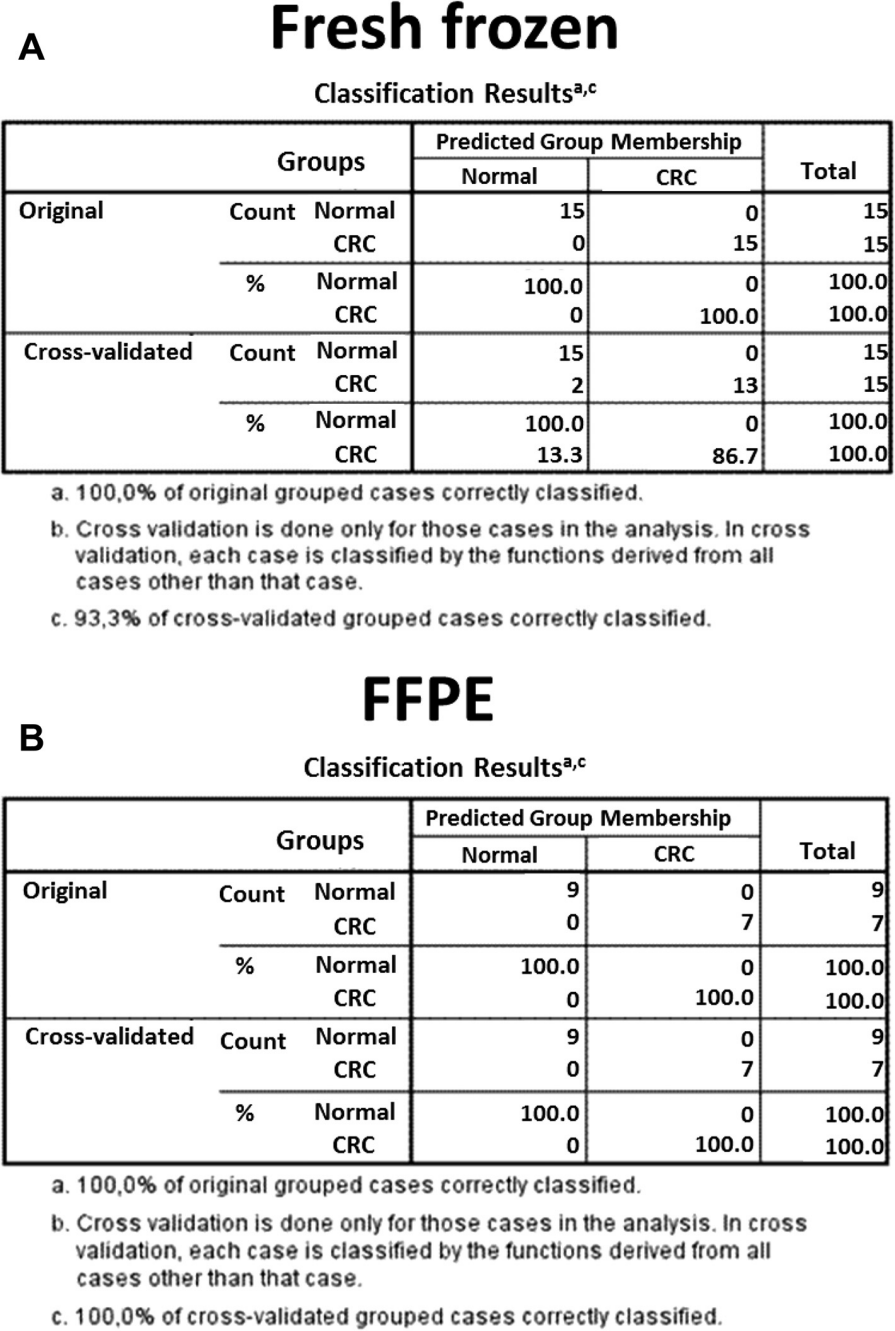
Discriminant analysis of (**a**) fresh frozen biopsy and (**b**) FFPE samples on the basis of gene expression levels of 11 transcripts. The table contains predicted group membership data on the original grouped cases and on the cross validated samples

Discrimination analysis was performed in order to test the classificatory power of the transcript set (Fig. 3) on independent fresh frozen biopsy and FFPE samples. In biopsy samples, the set could correctly classify the original grouped cases (100 %; 100 % respectively), while 93.3 % of cross validated grouped cases were correctly classified (Fig. 3a). From the FFPE sample set 6 normal and 8 tumor samples were automatically excluded in the discrimination analysis. The remaining 9 normal and 7 tumor samples could be classified correctly (100 %; 100 % respectively) (Fig. 3b).

### ROC analysis

**Fig. 4:**
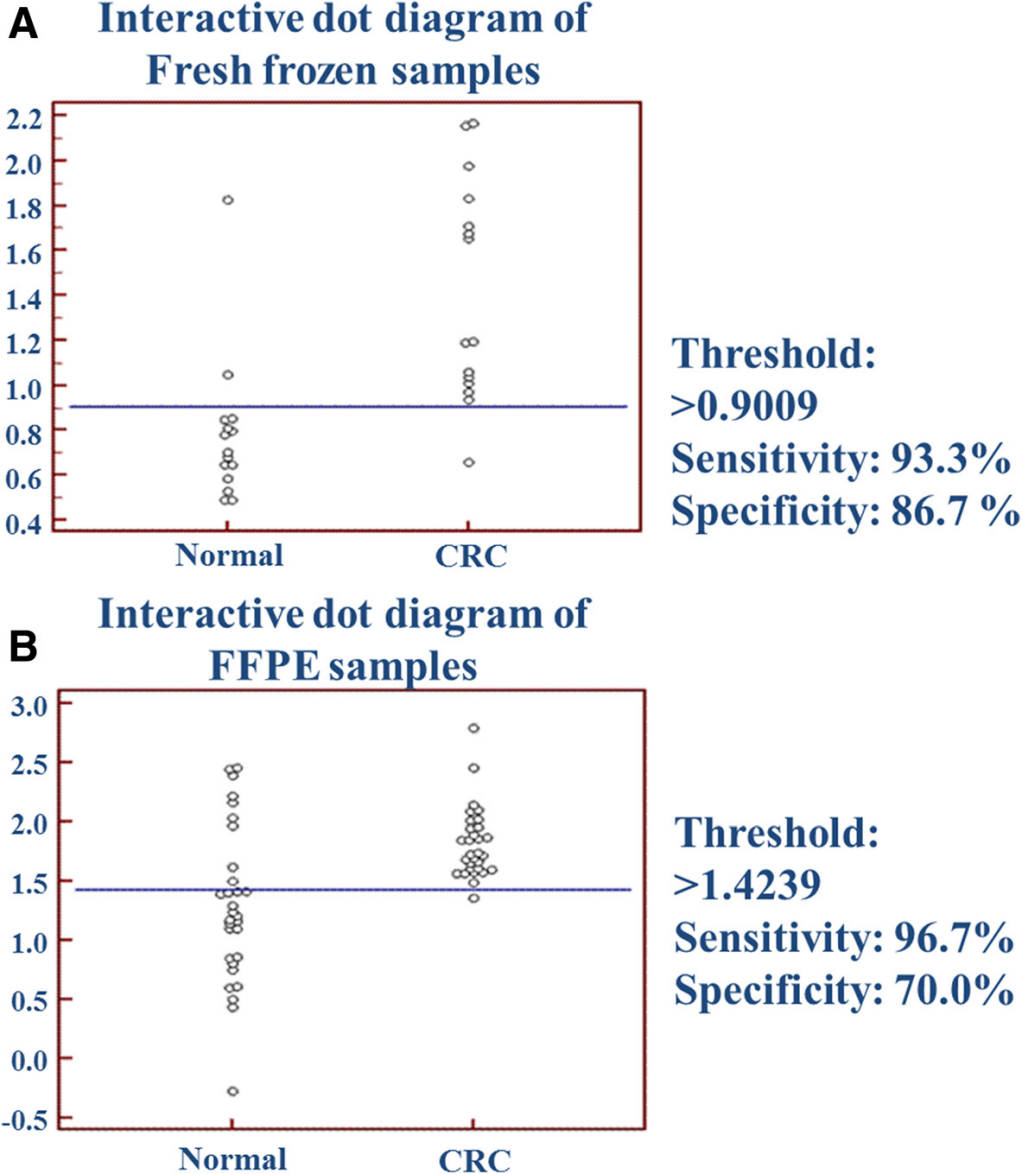
Interactive dot diagram of (**a**) fresh frozen biopsy and (**b**) FFPE samples according to the multiple logistic regression equation. On the basis of the previously published transcript set, a multiple logistic regression equation was applied to the present study’s results. The results can be visualized on interactive dotblots, normal and tumor groups are displayed as dots in two separate groups and the horizontal line indicates the cut off point with the best sensitivity and specificity results. The sensitivity and specificity values are calculated by the algorithm, that can be seen beside the dotblots

Paired comparison was performed with receiver operating characteristic (ROC) analysis in order to test the sensitivity and specificity of the transcript set on independent samples. Sensitivity and specificity were calculated from gene expression of the 11 discriminatory marker with the same equation that was established on the basis of multiple logistic regression on the original biopsy samples described in the recent manuscript about the marker set [2]. Normal and fresh frozen CRC samples could be distinguished by 93.3 % sensitivity and 86.7 % specificity (Fig. 4a). Normal and CRC FFPE tissue samples could be discriminated with higher specificity (96.7 %) and but with lower sensitivity (70.0 %) (Fig. 4b).

### In situ hybridization

**Fig. 5:**
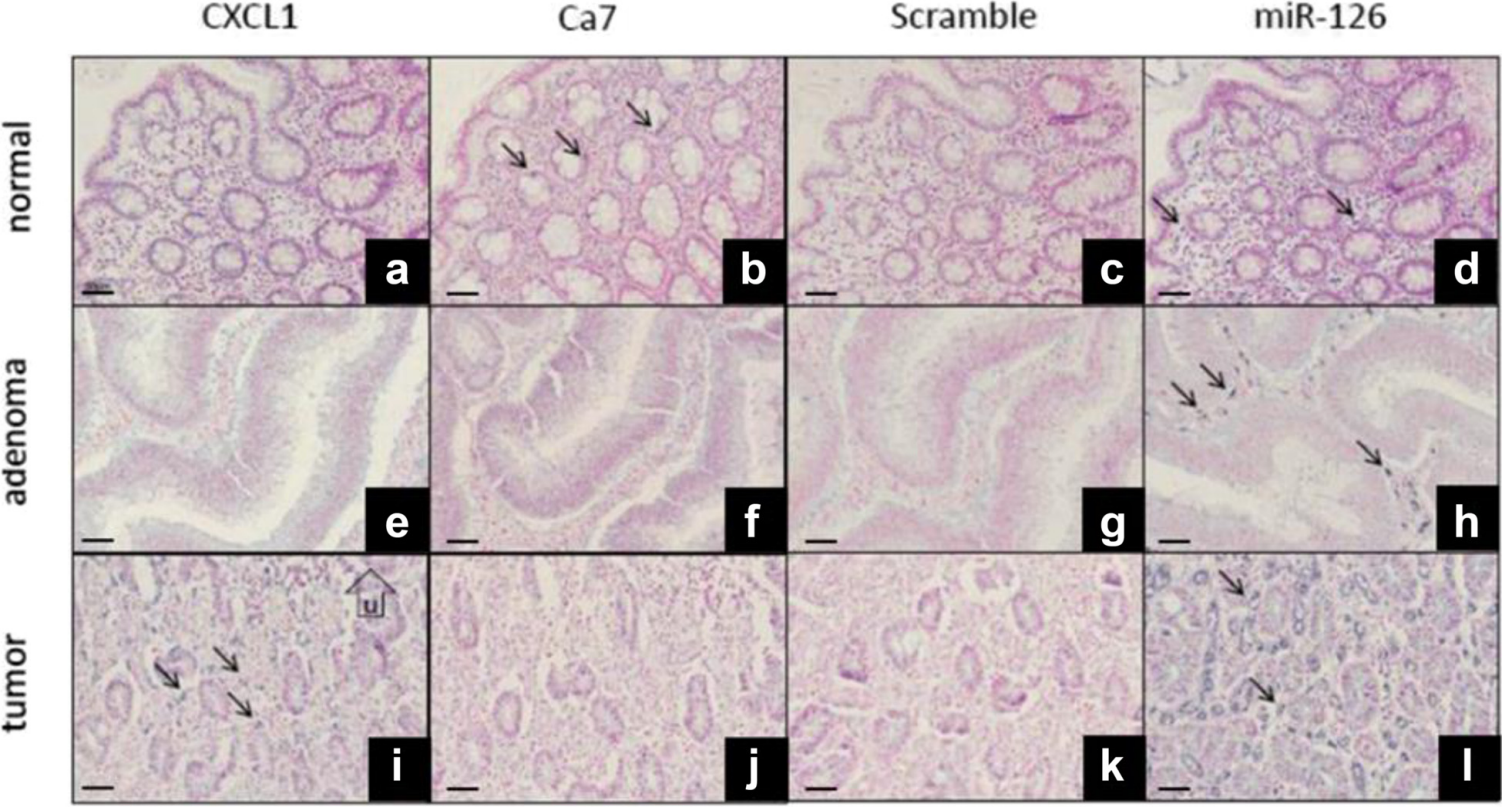
In situ hybridization for CA7 and CXCL1 mRNAs. Paraffin sections from normal colon (**a**-**d**), colon adenoma (**e**-**h**) and colon cancer (**i**-**l**) were stained with LNA probes for CXCL1 mRNA (**a**,**e**,**i**), CA7 mRNA (**b**,**f**,**j**), a generic unspecific sequence, scramble (**c**,**g**,**k**), and a positive control probe, miR-126 (**d**,**h**,**l**). The CXCL1 ISH signal is seen in a population of macrophage-like cells located in the cancer stroma (**i**, arrows) just below the luminal ulceration (indicated by U), whereas no CXCL7 ISH signal is detected in the normal colon mucosa and in the adenoma (**a**,**e**). The CA7 ISH signal is seen in a subset of epithelial cells in normal colon mucosa (**b**, arrows), whereas no ISH signal is detected in the colon adenoma and cancer tissue (**f**,**j**). miR-126 ISH signal is prevalent in endothelial cells (arrows in **d**,**h**,**l**), and only background staining is seen with scramble probe (**c**,**g**,**k**). Tissue sections were counter stained with Nuclear Fast Red. 50 μm bar is representative for all images

In situ hybridization was performed for CA7 and CXCL1, the two selected markers from the CRC-specific transcript set. CA7 was selected as the only downregulated transcript along CRC formation, while CXCL1 was included in the experiment, as it showed remarkable protein expression level changes between normal and CRC samples [10]. In a pilot experiment, the CA7 and CXCL1 probes were tested in various colon lesions. The probes incubated individually resulted in weak cellular staining with diffuse background, whereas the probes incubated in a mix resulted in a more robust signal with a better signal-to-noise ratio (data not shown). From three patients, we then examined paraffin samples from normal colon, adenoma and adenocarcinoma. All three FFPE adenocarcinoma specimens contained parts of luminal ulceration and invasive front. A weak distinct CA7 ISH signal was identified with the CA7 probe set in a subset of epithelial cells in all three normal colonic samples (Fig. 5). No CA7 ISH signal was detected in adenoma and colon cancer tissue. In the three cases of colon cancer examined, the CXCL1 ISH signal was seen in a subset of stromal cells located towards the central ulcer of the lesion (Fig. 5i). The stromal localization and the distribution pattern suggested that the CXCL1 mRNA-positive cells constitute a subset of macrophages. No CXCL1 ISH signal was detected in normal colon and in the colon adenomas.

## Discussion

Conventional tumor classification based on histopathology is likely to be accompanied by molecular biology methods in the future. In order to determine the applicability of a previously identified colorectal cancer-specific transcript set, gene expression of these markers were analysed on independent fresh frozen and also on FFPE samples. Furthermore, as methods enhanching diagnostic approaches are required to have straightforward workflow, automated RNA isolation and automated polymerase chain reaction setup were also integrated in the study.

The present study aimed to test the discriminatory power of a set of 11 transcripts. According to our results this mRNA transcript set could be suitably applied even on FFPE samples with relatively high sensitivity and specificity. Based on the expression level of the transcript set, independent fresh frozen biopsy samples could be classified correctly in original grouped cases (100 %; 100 %, respectively), while 93.3 % of cross validated grouped cases were correctly classified. The aim of this study was to confirm the possibility of discriminating FFPE samples by using mRNA markers. Although only a relatively low sample set was analyzed, an almost clear separation of normal vs. CRC FFPE samples could be achieved with unsupervised hierarchical clustering.

Analytical parameters of total RNA samples isolated from different sample sources can be critical regarding downstream molecular analyses. In our study yield of RNA isolated from FFPE and fresh frozen samples were found to be similar, which is in accordance with literature data [13]. In our analysis altogether 150 ng of total RNA was enough to analyze gene expression of 11 selected transcripts and an endogenous control on a custom made pre-lyophilized real-time PCR plate. As it can be expected according to previous methodical studies [14, 15], RNA purity ratios were found to be lower in RNA eluates isolated from FFPE samples and RIN was significantly higher (p < 0.001) in fresh frozen biopsies. However, the majority of the FFPE reactions had similar results as the fresh frozen samples and only three out of 11 assays showed higher presence of failed PCR reactions with FFPE samples. The use of even shorter amplicon sizes might result in successful reactions [16].

It is well known and documented, that colorectal cancer development is accompanied with gene expression alteration of several genes [17]. Many studies aimed to explore whole genome gene expression profiles of CRC cases attempting to identify clinically useful markers [18–20] and part of these projects focused on the discovery of altered gene expression patterns between normal colon mucosa and tumor tissue samples revealing different CRC-specific transcript sets [21–24]. Among the firsts, in 1999 Alon and collaborators identified a set of 2000 transcripts clustering 22 normal and 30 colorectal cancer tissue samples with highest minimal intensity across the samples [25]. Later, it was followed by subsequent studies reporting more restricted potential marker sets. For example, on the basis of Zou and collaborators’ results analyzing normal (n = 8) and CRC (n = 9 CRC) fresh frozen samples 250 differentially expressed transcripts could be identified [26]. Lin and colleagues examined normal (n = 20), adenoma (n = 9) and CRC (n = 11) fresh frozen tissue samples and could define 427 discriminatory markers [27]. Friederichs and collaborators identified 330 transcripts on the basis of gene expression data of CRC (n = 25) and normal (n = 6) colorectal fresh frozen tissue samples [28].

The comparison of gene expression profiling studies focusing on colorectal cancer development remains challenging. According to a recent systematic review of 23 independent expression analyses revealed only 54 genes, reported from at least two studies, showing the same expression alteration direction [29]. Furthermore, despite of the known molecular complexity underlying this disease, studies have been largely focused on the identification of single markers (e.g. upregulated IL8, AXIN2, CXCL12, CDK8; downregulated CA2, COL1A2, FABP1, IGFBP7) [30].

However, in order to achieve higher sensitivity and specificity multimarker sets should be analyzed, these are considered to be superior to single marker discriminations (Grabowski [31]). The sensitivity and the specificity of the transcript set [2] published by Galamb and colleagues in 2012 were assessed by discriminant analysis revealing 93.3 % sensitivity and 86.7 % specificity in fresh frozen biopsies and 96.7 % sensitivity and 70 % specificity in FFPE samples.

On the basis of Garcia-Bilbao and collaborators work published in 2012, relatively higher discriminating power of non-tumoral vs. CRC fresh frozen biopsy samples could be achieved on the basis of a set of 7 transcripts (ENC1, ACAT1, TMEM132A, CMTM7, FAM60A, MADCAM1, DDX55) with RT-qPCR with 90.9 % sensitivity and 100 % specificity [32]. Another study reported 83.3 % specificity and 70.8 % sensitivity in tumor vs. normal FFPE tissue classification by a prognostic marker set (with not only mRNA markers: neuroendocrine differentiation, overexpression of the sialyl-Lex antigen, overexpression of the peripheral benzodiazepine receptor (PBR), BAX protein expression and p53 mutational status) [31].

In situ analysis of two transcripts of the analyzed set showed the same gene expression alteration tendency, as found in RT-PCR experiments. CA-7 was found to be downregulated showing weak CA7 ISH signal in normal colon and no detectable signal in the analyzed adenomas and colon cancer FFPE tissue samples. In contrast, chemokine (C-X-C motif) ligand 1 was found to be upregulated according to our RT-PCR results. With in situ mRNA hybridization CXCL1 was also found to be an upregulated transcript in colorectal cancer FFPE tissues, with no CXCL1 ISH signal in the healthy and adenomatous colon tissue specimens. Interestingly, CXCL1 transcript could be localized in stromal cells towards the ulcerous areas of the tumors.

The ratio of failed reactions with FFPE samples were more abundant with some assays (MMP3, CA7, IL8) (Additional file 1: Table S1), that might be due to technical reason, e.g. relatively longer amplicon sizes (MMP3: 110 bp; IL8: 92 bp; Table 1) compared to the other assays or the presence of contaminants in RNA samples isolated from FFPE tissue samples. Furthermore, failed reactions might indicate biological reason in the background, e.g. possible downregulation of CA7 in CRC samples and low MMP3 expression in normal colon samples.

The majority of the transcripts (10 from the 11 markers) analyzed in this study was found to be upregulated along colorectal cancer formation also in fresh frozen and in FFPE tissue samples. On the other hand, gene expression alterations observed in our RT-PCR experiments of the 11 transcripts have already been reported in the literature. Interleukin 1 beta (IL1B) is proven to promote the invasiveness of malignant cells and this pro-inflammatory cytokine is required for angiogenesis [33]. The -31 T/C polymorphism of this gene is associated with colorectal cancer risk, as IL-1B-511 heterozygotes and T carriers has reduced risk of colorectal carcinoma development [34] and it is associated with the recurrence of CRC [35]. IL-1B is involved in the survival and proliferation of remnant cancer cells after tumor resection in colorectal carcinoma [36]. IL1RN polymorphism is also associated with CRC [37].

Interleukin 8 (IL8) overexpression has been documented in CRC; it is involved in proliferation, metastasis and angiogenesis, furthermore its knock down can inhibit colorectal cancer cell growth and metastasis formation [38, 39]. Gremlin -1 overexpression has been detected in various cancer types including pancreas, breast, kidney, ovary and colorectal cancer [40]. Secretion of gremlin-1 protein can enhance tumorigenesis via enhancing cell proliferation [41]. CXCL2 (alias Gro2) shows elevated expression in colorectal cancer already in premalignant lesions and in CRC [42] and it could accelerate tumor cell growth by inducing cell proliferation in a mouse implantation model [43]. Collagen type XII (COL12A1) was recently identified as a marker of myofibroblastic differentiation in CRC in the desmoplastic invasion fronts of the tumors [44]. Its overexpression could be detected in CRC-associated CAF (cancer-associated fibroblast) cells isolated from a mouse model of human sporadic cancer [45]. Chitinase 3-like 1 (CHI3L1, alias HCgp39 or YKL-40) is a secreted glycoprotein that is not synthesized physiologically, but in inflammatory and cancerous states [46, 47], it proved to have an important role in macrophage recruitment and angiogenesis during colorectal cancer formation [48]. According to a recent report, this marker can be usefully utilized in monitoring CRC-patients in the follow-up phase, as elevated serum YKL-40 levels is associated with short survival time [49]. Solute carrier family 7, member 5 was found to be upregulated 3-fold in the adenoma and 5-fold in CRC cases on the protein level [50]. Carbonic anhydrase 7 (CA7) plays role in acid-base balance, bone restoration, respiration, calcification and catalyze zinc^2+^ ion dependent hydration of CO_2_ [51]. According to a recently published manuscript from Yang and collaborators analyzing FFPE (n = 379) and fresh frozen CRC (n = 84) tissue samples, CA7 was frequently downregulated in tumor samples both on mRNA and protein levels. Furthermore, authors could correlate decreased CA7 expression levels with shorter disease-free survival time [52]. Downregulation of CA7 transcript along tumor formation and the presence of a large CpG island in the gene’s promoter region raises the theoretical possibility of its epigenetic gene regulation by DNA methylation; further studies are needed to confirm this hypothesis. According to the in situ hybridization results the extent of CA7 expression level alteration was lower than observed in RT-PCR experiments. However, CA7 expression levels were confirmed to be different between healthy and diseased colon samples according to the RT-PCR results.

CXCL1 (Groα) gene encodes a secreted interleukine-like molecule binding specifically to G-protein-coupled receptor CXCR2 [53] and potentially plays role in tumor-associated angiogenesis in non-small cell lung cancer [54]. Its overexpression has been described in melanoma [55] and in CRC on mRNA level with well-correlating immunohistochemical results [56, 57]. In 2015, le Rolle and colleagues found that CXCL1 overexpression is a poor prognostic marker on metastatic CRC, furthermore, CXCL1 inhibition could suppress tumor cell growth of KRAS mutant CRC cells [58]. On the basis of Oladipo and collaborators’ immunohistochemistry results of CRC FFPE samples (n = 254), CXCL1 protein level was significantly elevated in tumor tissue samples compared to normal adjacent tissue samples [59]. Matrix-metalloproteinase-3 (MMP3) is highly expressed in colorectal carcinomas [60], the encoded protein plays role in tumor invasion, lymph node involvement and metastatic spread [61].

## Conclusion

Automated nucleic isolation protocols can hold possibilities in gene expression studies due to the fact, that several samples can be processed in parallel in a highly standardized manner with minimal hands-on time. With the MagNA Pure 96 system high RNA amounts could be isolated from biopsies as well as from FFPE tissue slides. Although the quality of the FFPE samples were found to be lower compared to the fresh frozen material, in combination with pre-optimized short amplicon sized assays, this sample type could also serve similar results as fresh frozen biopsy samples. The set could discriminate between normal colonic and CRC FFPE samples with 96.7 % sensitivity and 70.0 % specificity. According to our results, gene expression analysis of the analyzed colorectal cancer-specific marker set can be performed from FFPE tissue material. With the addition of an automated workflow, this marker set may enhance the objective classification of colorectal neoplasias in the routine procedure.

## Additional file

Additional file 1: Table S1. Ratio of successful/failed real-time polymerase chain reactions (RT-PCR) of the analyzed markers.

## Abbreviations

FFPE: Formalin-fixed, Paraffin-embedded
CRC: colorectal cancer
NAT: Normal adjacent tissue
RIN: RNA Integrity Number
ROC: Receiving operating characteristic analysis
