# Supplementary material for: Gene-expression analysis of a colorectal cancer-specific discriminatory transcript set on formalin-fixed, paraffin-embedded (FFPE) tissue samples
Source: Diagn Pathol. 2015 Jul 25;10:126. doi: 10.1186/s13000-015-0363-4 (PMC4515026; doi:10.1186/s13000-015-0363-4)
Supplement: Additional file 1: Table S1. — Ratio of successful/failed real-time polymerase chain reactions (RT-PCR) of the analyzed markers. [file 13000_2015_363_MOESM1_ESM.pdf]

Gene symbol	Gene name	Number of successful / failed RT-PCR reactions			
		Fresh frozen samples		FFPE samples	
		Normal samples	CRC samples	Normal samples	CRC samples
**CA7**	carbonic anhydrase VII	15/0	15/0	15/0	7/8
**CHI3L1**	chitinase 3-like 1	15/0	15/0	15/0	15/0
**COL12A1**	collagen, type XII, alpha 1	15/0	15/0	15/0	15/0
**CXCL1**	chemokine (C-C-C motif) ligand 1	15/0	15/0	14/1	15/0
**CXCL2**	chemokine (C-C-C motif) ligand 2	15/0	15/0	15/0	15/0
**GREM1**	gremlin 1	15/0	15/0	15/0	15/0
**IL1B**	interleukin 1, beta	15/0	15/0	15/0	15/0
**IL1RN**	interleukin 1 receptor antagonist	15/0	15/0	15/0	15/0
**IL8**	interleukin 8	15/0	15/0	13/2	15/0
**MMP3**	matrix metallopeptidase 3	15/0	15/0	9/6	15/0
**SLC7A5**	solute carrier family 7, member 5	15/0	15/0	15/0	15/0
**RN18S1**	RNA, 18S ribosomal 1, 18S ribosomal RNA	15/0	15/0	15/0	15/0
